# A Macrophage/Monocyte‐Related Four‐Gene Signature for Prognostic Assessment of Uveal Melanoma: *BTBD6*, *C2CD4B*, *CCL24*, and *S100A4*

**DOI:** 10.1155/humu/4978880

**Published:** 2026-06-15

**Authors:** Yuhui Yu, Mengya Wang, Bin Zhao

**Affiliations:** ^1^ Department of Ophthalmology, The Second Affiliated Hospital of Shandong First Medical University, Tai′an, Shandong, China, natureindex.com; ^2^ Department of Anorectal Surgery (TCM), The People′s Hospital of Feicheng City, Feicheng, Shandong, China

**Keywords:** immunity, macrophage, monocyte cells, prognosis, uveal melanoma, WGCNA

## Abstract

**Background:**

Uveal melanoma (UVM) is a highly malignant ocular tumor with a poor prognosis. Macrophages and monocytes in the tumor microenvironment promote immune escape, angiogenesis, and metastasis. Thus, exploring their roles may provide insights into UVM progression.

**Methods:**

The Cancer Genome Atlas (TCGA) was accessed to obtain the data of mRNA expression and follow‐up data of UVM, and UVM single‐cell profiles were downloaded to cluster cells by annotation of single‐cell marker genes. The differentially expressed genes (DEGs) in macrophage/monocyte cells compared to other cell types were revealed. ssGSEA was applied to compute the score of DEGs and to reveal the genes for WGCNA in UVM. A prognostic risk model for UVM was constructed by uni/multivariate Cox and LASSO regression analyses to reveal the differential overall survival status. Further cellular validations were conducted to examine the effects of core genes in UVM. TIMER tool was applied for the analysis of immune cell infiltration levels in UVM. Chemotherapeutic drug sensitivity in UVM was assessed with the pRRophetic package.

**Results:**

Six cell subpopulations were identified in the UVM samples, among which macrophage/monocyte cells were more predominant. Kaplan–Meier curves showed that UVM patients in the group of high RiskScore (consisting of the genes *BTBD6*, *C2CD4B*, *CCL24*, and *S100A4*) presented a poorer prognosis, higher infiltration of monocytic lineage, T cells, CD8 T cells, cytotoxic lymphocytes, and higher expression of immune checkpoint–related genes. A significant negative correlation between RiskScore and the IC_50_ of XMD8‐85, lapatinib, roscovitine, salubrinal, bexarotene, LFM‐A13, FTI‐277, and TGX221 chemotherapeutic agents was further noticed.

**Conclusion:**

In this study, we computationally identified genes associated with both disease progression and macrophage/monocyte‐related characteristics in UVM and constructed a prognostic risk model with predicted immune infiltration patterns. These findings generate testable hypotheses that may inform future experimental studies on the immune mechanisms underlying UVM.

## 1. Introduction

Uveal melanoma (UVM) is a rare and highly malignant ocular tumor that occurs primarily in the uveal region of the eye [1, 2]. Approximately 50% of patients with UVM develop metastases within 10 years of diagnosis, and liver metastases from the cancer occur in approximately 90% of cases with confirmed metastatic lesions [3, 4]. Poor prognosis and poor early diagnosis of UVM result in an extremely high mortality rate for patients [5]. Moreover, metastatic UVM lacks effective systemic therapies and exhibits resistance to immune checkpoint inhibitors [6, 7]. Therefore, it is particularly important to propose effective diagnostic and therapeutic approaches. Currently, TNM staging is an effective method for detecting UVM staging, which is important for cancer prognosis and provides guidance for proper therapeutic approaches [8, 9]. However, the clinical limitations of the TNM staging method have gradually emerged with the deepening of tumor research, and some studies have revealed that there are also differences between the overall survival rates associated with the TNM staging method [10, 11]. Therefore, there is a need to explore new UVM markers for use in guiding clinical treatment and improving the prognosis of UVM.

Most components of the immune system are implicated in melanoma development [12–14]. The regulatory role of macrophages and monocyte cells in melanoma has gradually attracted attention. Relevant studies have shown that melanoma‐derived exosomes mediate immunosuppression and their regulatory mechanism is mainly through macrophage M2 polarization, which in turn promotes the recruitment of tumor regulatory T cells [15]. The regulatory role of monocytes on the immune response to UVM is mainly reflected by the fact that the cells phagocytose antigens and then transfer the antigenic determinants they carry to lymphocytes, influencing the immunogenic response of lymphocytes [16]. In addition, monocytes usually present an immunosuppressive phenotype, leading to the depletion of T cells and NK cells in the tumor microenvironment (TME). Such a process is revealed to be mediated by multiple mechanisms including reactive oxygen/nitrogen production, nutrient depletion, secretion of inhibitory cytokines, and expression of immune checkpoint ligands [17, 18]. In addition to the direct regulatory role of immune cells, immune escape of cancer cells is also an important factor in the development of UVM, and thus, PD‐1, PD‐L1, and CTLA‐4 inhibitors are often used in the treatment of clinical UVM, aiming to enhance the cancer eradication ability of the immune system in UVM patients by suppressing the immunosuppressive function of immune checkpoints [19]. Therefore, given the central role of the monocyte/macrophage axis in mediating immunosuppression and immune escape in UVM, it is urgent to systematically explore the association between monocyte/macrophage‐related genes and UVM prognosis, thereby identifying reliable biomarkers along this axis for prognostic assessment.

Here, we focused on the single‐cell map of UVM as the entry point, mined the key genes related to macrophage/monocyte cells through clustering analysis and weighted gene coexpression network analysis (WGCNA), and combined with various types of regression analyses to identify the biomarkers affecting the prognosis of UVM in order to establish a prognostic model of UVM. Both the construction of the risk assessment model and the subsequent investigation of the model in the regulation of the immune microenvironment of UVM will help to provide guidance for both the exploration of novel targets for UVM and the advances of clinical therapeutics.

## 2. Methods

### 2.1. Data Source

This study integrated UVM data, RNA sequencing, and clinical follow‐up data of 79 samples (TCGA‐UVM) obtained from The Cancer Genome Atlas (TCGA, https://www.cancer.gov/ccg/research/genome-sequencing/tcga) program [20], and the dataset GSE44295 contributed gene expression profiles for an additional 57 UVM samples acquired from the Gene Expression Omnibus (GEO, https://www.ncbi.nlm.nih.gov/geo/) database [21].

The single‐cell dataset for UVM GSE139829 was additionally downloaded from the GEO database, containing eight primary tumor samples and three metastatic tumor samples.

### 2.2. scRNA‐Seq Analysis

The function “CreateSeuratObject” of the Seurat package was first applied to create the Seurat object [22], and an initial filter was performed to retain only cells with a gene count between 200 and 8000 and a mitochondrial gene ratio of less than 10%. Next, the “SCTransform” function was used to normalize the data and to eliminate differences in sequencing depth, followed by principal component analysis (PCA) downscaling via RunPCA. Batch effect for different samples was removed by the “RunHarmony” function of the harmony package, setting lambda = 0.5 and max.iter.harmony = 50. The functions FindNeighbors and FindClusters were used for clustering at the parameters dims = 1 : 30 and resolution = 0.1, and UMAP was performed using the function “RunUMAP” for dimensionality reduction. Finally, cells were annotated based on the marker genes provided by the CellMarker2.0 database (http://117.50.127.228/CellMarker/). Differentially expressed genes (DEGs) were calculated for different cell types using the FindAllMarkers function.

### 2.3. Functional Enrichment Analysis

Leveraging the clusterProfiler package, we conducted functional enrichment analysis on the macrophage/monocyte‐derived DEGs to identify significantly enriched terms in Gene Ontology (GO) and pathways in Kyoto Encyclopedia of Genes and Genomes (KEGG) analysis [23].

### 2.4. Single‐Sample GSEA (ssGSEA)

In this study, we used the ssGSEA of the GSVA package [24] to compute the characterization scores of DEGs in macrophage/monocyte cells.

### 2.5. WGCNA

WGCNA package [25] was utilized to identify target modules closely associated with the ssGSEA score of DEGs in macrophage/monocyte cells. The pickSoftThreshold function was employed to determine the soft threshold *β*. Hierarchical clustering was then used to find gene modules under the criterion of minModuleSize = 60. The correlation between each module and the ssGSEA score was subsequently analyzed; the module with the most significant correlation was selected as the target module, and the genes characterizing the module therein were applied in the subsequent analysis.

### 2.6. Establishment of a Prognostic Risk Model and Validation

The prognostically relevant genes were initially screened using univariate Cox regression analysis, and the screening criterion was a *p* value of less than 0.01. Then, we further obtained the key genes and correlation coefficients that were highly correlated with the prognosis of UVM by LASSO Cox analysis and multivariate Cox regression analysis, and constructed a prognostic risk assessment model for UVM patients. The calculation formula is shown as follows:

RiskScore=∑βi∗expression i,

where *i* is the gene expression and *β* is the Cox regression coefficient of the gene.

### 2.7. Immune Cell Infiltration

The MCPcounter package [27] was used to compute immune cell scores in UVM samples from the high RiskScore and low RiskScore groups. The immune scores of the six cell types were computed by applying the TIMER online tool (http://cistrome.org/TIMER). The expression level of immune checkpoint genes was also analyzed in UVM samples from the low/high RiskScore groups.

### 2.8. Drug Sensitivity Analysis

The differential sensitivity to anticancer drugs between samples in the high/low RiskScore groups was explored based on the inhibitory concentration in half (IC_50_) of chemotherapeutic agents used for the treatment of UVM via the pRRophetic package [28], and a correlation between IC_50_ and RiskScore was thereafter established.

### 2.9. Cell Culture and Transfection

Melanoma cell line A375 (CRL‐1619) and human epidermal melanocytes (PCS‐200‐013) were purchased from American Type Culture Collection (ATCC, Manassas, Virginia) and cultured as follows. The melanocytes were cultured in the specified growth kit (PCS‐200‐042, ATCC), and A375 cells were grown in DMEM (30‐2002, ATCC) added with 10% bovine calf serum (30‐2020, ATCC). All cells were maintained in an incubator at 37°C with 5% CO_2_.

For liposome transfection, the small interfering RNAs targeting C2CD4B or the scramble control without the specified target sites were ordered from RiboBio (Guangzhou, China) and transfected into A375 cells using Lipofectamine 2000 transfection reagent (11668027, Invitrogen, Carlsbad, California). The detailed sequences of relevant siRNAs are shown in Supporting Information 1: Table S1.

### 2.10. Quantitative Reverse Transcription PCR (qRT‐PCR)

The total RNA samples from cells were extracted using TRIzol (15596‐026, Invitrogen) reagent, and the concentration was tested. The corresponding cDNA was synthesized with a corresponding synthesis kit (D7178S, Beyotime, China), and the PCR was performed in the real‐time PCR thermocycler (X320, Heal Force, Shanghai, China) using the SYBR Green qPCR Mix (D7260, Beyotime, China). The 2^−*ΔΔ*CT^ method was applied for the quantification, and the housekeeping control GAPDH was applied for normalization [31]. The sequences of primers used are referred to in Supporting Information 2: Table S2.

### 2.11. Scratch and Transwell Assays

A six‐well plate with nonserum media was utilized to grow transfected A375 cells, and a man‐made scratch was created with a 200‐*μ*L sterile pipette tip on the monolayers once the cells reached complete confluence. After 48 h, an inverted optical microscope (Olympus, Tokyo, Japan) was employed to photograph the cells and measure the wound closure degree (%).

The cell invasion assay was implemented using a 24‐well Transwell plate with a polycarbonate membrane (pore size: 8 *μ*m, 3422, Corning Inc., Corning, New York) coated with the prethawed matrix gel (C0372, Beyotime, China). Transfected A375 cells in 200 *μ*L of serum‐free media were cultured in the upper chamber, while 700 *μ*L of culture media containing 10% serum was added to the corresponding bottom chamber. After 48 h, the invaded cells were fixed and colored with 0.1% crystal violet (C0121, Beyotime, China) for 30 min, and three random fields were applied to quantify the number of invaded cells in the inverted optical microscope (Olympus, Japan) [29].

### 2.12. Statistical Analyses

R software (Version 3.6.0) and GraphPad Prism (Version 8.0.2) were used to analyze the data of computational and laboratory analyses. The difference between continuous variables in the two groups was determined via the Wilcoxon rank‐sum test, and the difference in survival time between each group of patients was compared based on the KM curves and the log‐rank test. The analysis of variance followed by Bonferroni′s multiple comparisons test was applied for the comparison of the experimental data. The threshold of *p* value < 0.05 was established when the data were statistically significant.

## 3. Results

### 3.1. Single‐Cell Mapping of UVM

**Figure 1:**
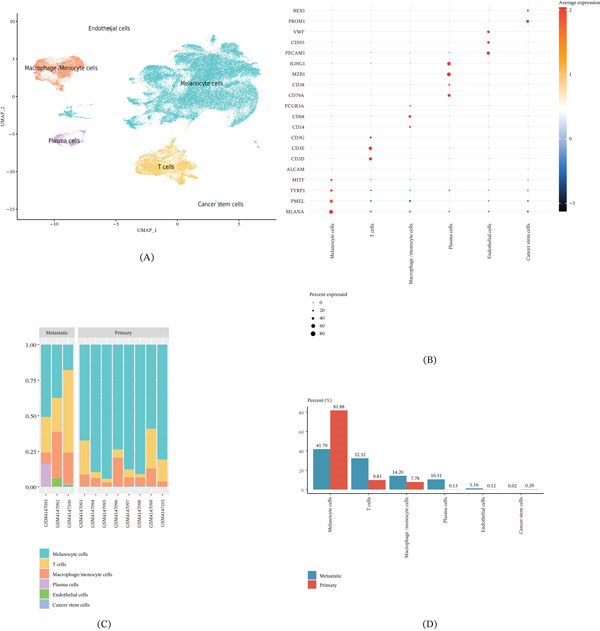
Single‐cell mapping of UVM. (A) Cell‐annotated UMAP plot after clustering of individual cells in UVM. (B) Marker gene expression in cell subpopulations in UVM. (C) Percentage of cells in individual UVM samples. (D) Comparison of cell types in primary UVM and metastatic UVM samples.

Single‐cell cluster annotation analysis was performed on primary UVM samples and metastatic UVM samples, and a total of six cell subpopulations were annotated, namely, plasma cells, melanocyte cells, T cells, macrophage/monocyte cells, endothelial cells, and cancer stem cells (Figure 1A). The marker genes for these cell subpopulations are shown in Figure 1B. The infiltration levels of these six cell subpopulations in each UVM sample showed differences, with a higher percentage of melanocyte cells in the primary UVM group (Figure 1C). In addition, the percentage of macrophage/monocyte cells was higher in the metastatic UVM group than in the primary UVM group (Figure 1D).

### 3.2. Functional Enrichment Analysis

**Figure 2:**
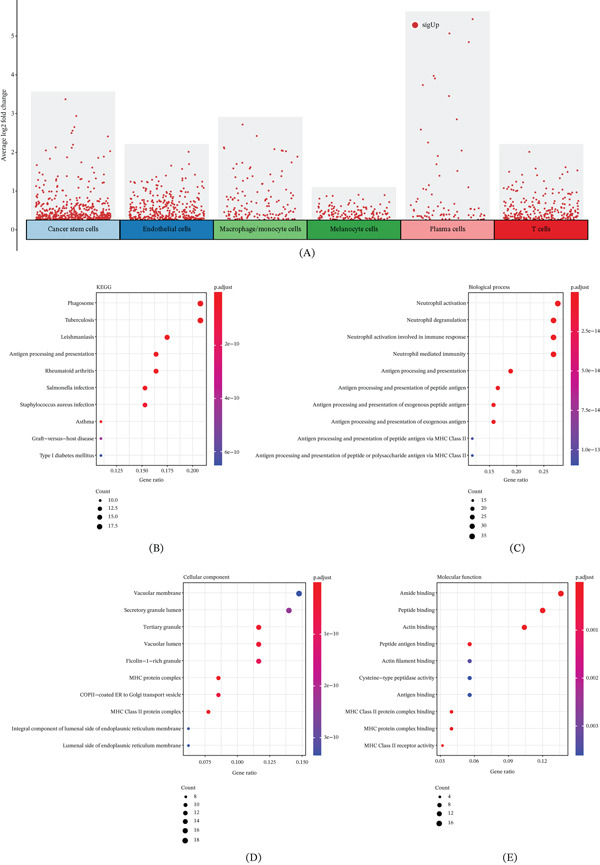
Identification of genes characterizing macrophage/monocyte cells in UVM. (A) Differentially expressed genes among cell subpopulations in UVM. (B–E) DEGs in the cell subpopulation of macrophage/monocyte cells subjected to GO and KEGG enrichment analysis.

The heterogeneity of gene expression patterns among cell subpopulations was then explored, and the DEGs of each cell subtype were analyzed (Figure 2A). KEGG enrichment analysis of DEGs of macrophage/monocyte cells showed that these genes were mainly enriched in antigen processing and presentation and phagosome (Figure 2B). These genes were mainly enriched in the biological processes of neutrophil activation and antigen processing and presentation of peptide antigen via MHC Class II (Figure 2C), the cellular components of secretory granule lumen and MHC protein complex (Figure 2D), and the molecular functions of amide binding and MHC Class II protein complex binding (Figure 2E).

### 3.3. WGCNA Mining of Genes Associated With Macrophage/Monocyte in UVM

**Figure 3:**
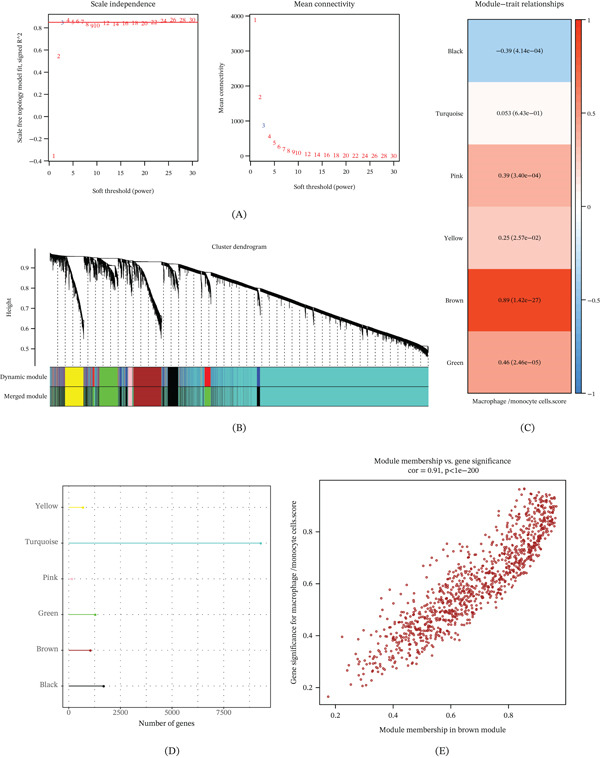
WGCNA for UVM. (A) Scale‐free fit index analysis for various soft threshold powers (*β*) and various soft threshold powers in average connectivity analysis. (B) Gene dendrogram based on 1‐TOM. (C) Correlation of the modular eigenvectors of each module with the macrophage/monocyte cell score. (D) The number of genes in each gene module identified via WGCNA. (E) Correlation of module membership (MM) in the brown feature module with macrophage/monocyte cell score.

In this study, WGCNA was performed on UVM samples to cluster genes to better identify gene modules associated with macrophage/monocyte cell score. The soft threshold *β* = 3 was selected for a scale‐free network (Figure 3A). Gene modules were identified using hierarchical clustering, which produced six coexpression modules after merging the modules (Figure 3B). Subsequently, this study further analyzed the association of each module with macrophage/monocyte cell score, revealing a strong correlation between the brown module and macrophage/monocyte cell score (Figure 3C). Follow‐up studies examined the genes in the brown module as module signature genes highly correlated with macrophage/monocyte cells (Figure 3D,E).

### 3.4. Construction of a Prognostic UVM Risk Model

**Figure 4:**
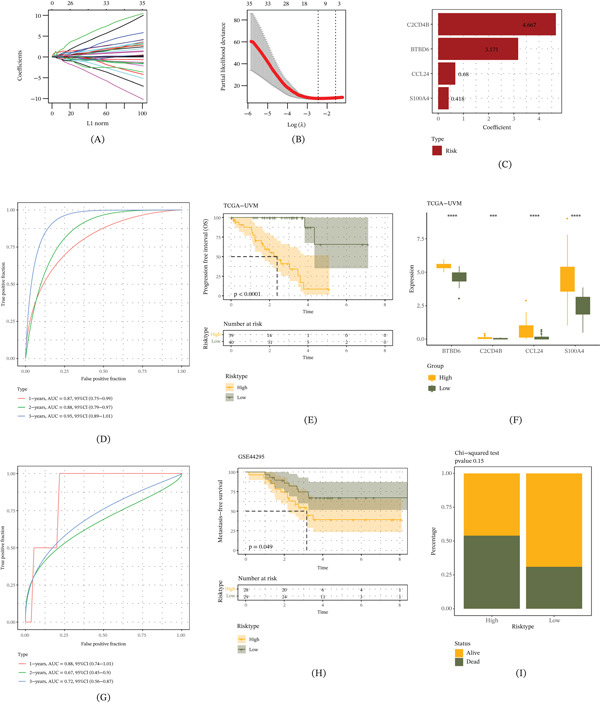
Construction and validation of the UVM prognostic risk model. (A) Gene coefficients generated with log(*λ*) in the LASSO model. (B) LASSO coefficient profiles of genes obtained from the LASSO regression. (C) Distribution of coefficients of prognostic‐related genes. (D) ROC curves of RiskScore in the TCGA training set data. (E) The survival curves in the TCGA training set. (F) Relative expression levels of prognostic genes in the TCGA training set. (G) ROC curves of RiskScore in the GSE44295 dataset. (H) KM survival curves in the GSE44295 dataset. (I) Survival status distribution of the patients in the two risk groups in the GSE44295 dataset.  ^∗∗∗^ *p* < 0.01 and  ^∗∗∗∗∗^ *p* < 0.001.

The modular signature genes screened by WGCNA were further subjected to univariate Cox and LASSO regression analyses using 10‐fold cross‐validation to improve the generalization ability of the model (Figure 4A,B). Subsequently, multivariate stepwise regression analysis determined four prognostically relevant genes for the prognostic risk model (Figure 4C):

RiskScore = 3.171∗*B* *T* *B* *D*6 + 4.667∗*C*2*C* *D*4*B* + 0.68∗*C* *C* *L*24 + 0.418∗*S*100*A*4.

TCGA‐UVM samples were then categorized into low‐risk and high‐risk groups by RiskScore. The model exhibited AUC values of 0.87, 0.88, and 0.95 for predicting 1‐, 2‐, and 3‐year survival status of UVM patients, respectively (Figure 4D). KM curve demonstrated that high‐risk UVM patients had a poorer prognosis (Figure 4E).

The expressions of the four prognosis‐related genes in the samples from the high‐ and low‐risk groups also showed significant differences, and they all showed significantly upregulated expression in high‐risk patients (Figure 4F). To validate the stability and reliability of our constructed UVM prognostic model, we utilized the GSE44295 dataset as an independent validation set. The RiskScore of patients was calculated using the same methodology as TCGA‐UVM, and the validation results were in accordance with the training set findings that patients with high‐risk scores had a worse prognosis (Figure 4G,H). In the GSE44295 validation cohort, the high‐risk group had a higher percentage of death samples (Figure 4I). Taken together, these results support the utility of the risk assessment model in predicting clinical outcomes for UVM.

**Figure 5:**
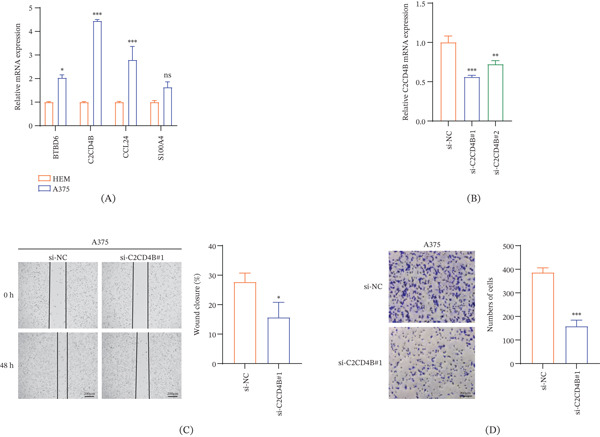
Role of four prognostically relevant genes. (A) Quantitative reverse transcription PCR for the calculated levels of four prognostically relevant genes in human epidermal melanocytes. (B) Validation of the knockdown efficiency of C2CD4B. (C, D) Scratch and Transwell assays exploring the migration and invasion of A375 cells in vitro.  ^∗^ *p* < 0.05;  ^∗∗^ *p* < 0.01;  ^∗∗∗^ *p* < 0.001; ns, *p* > 0.05.

Then, the involvement of four prognosis‐related genes in UVM was determined using in vitro cultured A375 cells. The data of PCR first demonstrated the evidently higher levels of all four genes in A375 cells (Figure 5A). Considering the relatively higher level of C2CD4B, we further explored its effects on A375 cells, and the obvious decreased mRNA level indicated the successful knockdown (Figure 5B). The results of scratch (Figure 5C) and Transwell (Figure 5D) have further demonstrated that C2CD4B knockdown in vitro repressed migration and invasion of A375 cells (Figure 5C,D).

### 3.5. Immune Cell Infiltration Differences

**Figure 6:**
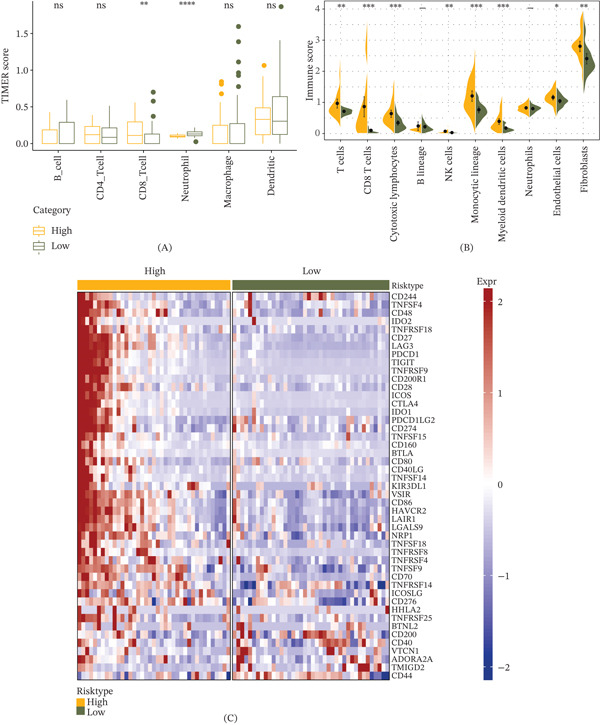
Immune cell infiltration in UVM. (A) Immune cell infiltration calculated based on the TIMER method. (B) MCP counter evaluated 10 immune cell scores. (C) Expression of immune checkpoint–related genes (^****^ *p* <0.0001, ^***^ *p* <0.001, ^**^ *p* <0.01, ^*^ *p* <0.05).

In TCGA‐UVM, the TIMER algorithm revealed that CD8 T‐cell scores were higher in the high RiskScore group, whereas the opposite was true for neutrophil (Figure 6A). In addition, the results of immune cell scores calculated using the MCP algorithm revealed that T cells, CD8 T cells, cytotoxic lymphocytes, and monocytic lineage scores were higher in the high RiskScore group than in the low RiskScore group (Figure 6B).

The expression of immune checkpoint genes was also analyzed in this study. The high RiskScore group showed higher expression of multiple immune checkpoint genes, including *CD244*, *TNFSF4*, *CD48*, and *PDCD1* (Figure 6C). This demonstrated a correlation between the RiskScore constructed in this study and immune cell infiltration and immune checkpoint expression in UVM, suggesting that the prognostic signature genes might regulate the progression of UVM by mediating tumor immune escape.

### 3.6. Correlation of RiskScore and Drug Sensitivity in UVM

**Figure 7:**
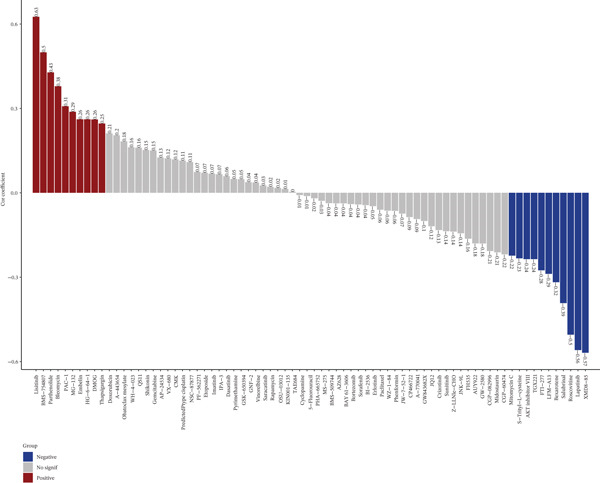
Correlation of IC_50_ values of various chemotherapeutic agents with risk scores in TCGA‐UVM samples.

The relationship between RiskScore and conventional chemotherapeutic drugs in TCGA‐UVM was explored. It was visible that the IC_50_ of chemotherapeutic drugs like XMD8‐85, lapatinib, roscovitine, salubrinal, bexarotene, LFM‐A13, FTI‐277, and TGX221 chemotherapeutic agents was significantly negatively correlated with the RiskScore, suggesting that UVM samples with higher RiskScore were more sensitive to these agents (Figure 7).

## 4. Discussion

UVM is the most prevalent intraocular malignancy and the leading lethal ocular disease in adults, but most are asymptomatic in the early stages and susceptible to distal metastasis. Currently, immunotherapy is an effective treatment option for melanoma patients [30]. Many researches have also demonstrated that immune components of the tumor immune microenvironment can be used to assess patient prognosis [31–33]. In this study, by integrating single‐cell transcriptomic data with bulk transcriptomic data, we identified four key genes—*BTBD6*, *C2CD4B*, *CCL24*, and *S100A4*—from the macrophage/monocyte‐associated gene set and used them to construct a prognostic risk model for UVM. This model effectively distinguished high‐risk from low‐risk patients in both the training and validation sets. The high‐risk group exhibited poorer survival outcomes, higher immune checkpoint expression, and distinct drug sensitivity profiles. These findings provide new candidate biomarkers for immune‐related prognostic assessment in UVM and lay a theoretical foundation for subsequent functional validation and the exploration of personalized treatment strategies.

The potential biomarkers of UVM mined in this study included *BTBD6*, *C2CD4B*, *CCL24*, and *S100A4*. Among them, *BTBD6* is one of the members of the BTB family, which is potentially linked to the progression of cancer, and the existing studies mined the immune cell infiltration, apoptosis, and glycolysis of the gene in melanoma and breast cancer in associated diseases by bioinformatic tools characterization [34, 35]. *C2CD4B* acts as an inducer of cellular oxidative stress in existing reports and regulates PI3K/Akt/PKC*α* pathway activity [36]. The activation of the PI3K/Akt signaling pathway is implicated in cancer cell survival and proliferation, which directly correlates with cancer progression and is thus recognized as an important target in cancer therapy [37]. In addition, the activation of PI3K/Akt can regulate macrophage activation and M1/M2 phenotype [38]. This is consistent with the results here, as seen in the discovery that the tapped *C2CD4B* is a gene potentially associated with macrophage features. The *CCL24* was reported in the available reports to regulate *CCR3* to modulate M2 macrophage polarization and fibroblast activation [39]. This has important implications for the exploration of the treatment and progression mechanisms of cancers such as UVM. Related studies have also demonstrated that this molecule is upregulated in the cancer immune microenvironment and affects tumor response to immune checkpoint inhibitors [40]. *S100A4* is mainly derived from immune cells, and the molecule exerts immunomodulatory functions in cancer and chronic inflammation [41–43]. In terms of regulatory mechanisms, *S100A4* can modulate macrophage polarization phenotype by regulating PPAR‐*γ*‐dependent fatty acid oxidation induction, which in turn affects macrophage protumorigenic activity [44]. Collectively, although direct functional evidence in UVM is lacking, these four genes have each been linked—through distinct pathways—to macrophage/monocyte biology, immune modulation, or critical oncogenic signaling. Their convergent association with immune‐related processes supports the biological plausibility of the proposed prognostic signature and warrants future experimental investigation into their integrated roles within the UVM TME.

The UVM samples were classified into high‐ and low‐risk groups as per the calculated RiskScore, and immune infiltration analysis revealed the differences in immune cell infiltration levels between the groups, in which the high‐risk group displayed a higher score of CD8_T cells, whereas neutrophil scored the opposite. CD8^+^ T cells are usually an important element of antitumor immunity, and their high infiltration levels are usually associated with a better cancer prognosis [45]. However, in some cases, increased CD8^+^ T‐cell infiltration in high‐risk groups might be associated with immune escape mechanisms. For example, CD8^+^ T cells can lead to immune cell depletion by upregulating the expression of immune checkpoint molecules PD‐1 (*PDCD1*), CTLA‐4 (*CD152*), and TIM‐3 (*HAVCR2*), leading to the inability of highly infiltrated CD8^+^ T cells to effectively kill the tumor cells [46–48]. Neutrophil function in cancer progression is heterogeneous, primarily manifesting as either an antitumor (N1) or a protumor (N2) phenotype; this polarization is determined by specific cytokines (e.g., IFN‐*β* and IL‐1*β*) within the TME after recruitment [49]. In the immune microenvironment of melanoma, a related study based on mouse experiments and clinical tissue assays identified extensive neutrophil activation in melanoma tissues treated with immune checkpoint blockade, and further experiments confirmed that the addition of immune checkpoint inhibitors enabled the production of antitumor neutrophil subpopulations in the melanoma immune microenvironment [50, 51]. Together, these findings imply that the macrophage/monocyte‐related prognostic genes may promote UVM progression by modulating both immune cell activity and tumor cell immune evasion.

Nonetheless, some inevitable shortcomings exist in the present study. For example, as a purely computational analysis, the direct regulatory relationships between the four identified key genes (*BTBD6*, *C2CD4B*, *CCL24*, and *S100A4*) and macrophage/monocyte biology and immune pathways lack experimental validation. Future studies should establish in vitro coculture models and assess changes in macrophage polarization markers and downstream immune factors to clarify their regulatory roles. Second, the association between the prognostic model and immune infiltration and drug sensitivity is based solely on transcriptomic data from public databases and has not yet been validated in clinical UVM tissue samples. In future studies, tumor tissue from an independent UVM cohort should be collected to validate the correlation between gene expression in the model and the infiltration levels of specific immune cells (such as CD8^+^ T cells) using immunohistochemistry and multiplex immunofluorescence. Finally, since this study did not involve in vivo experiments, future research should establish a mouse model of UVM xenograft tumors. By knocking out or overexpressing key genes in these models, we can assess changes in tumor growth, metastasis, and the immune microenvironment, thereby providing direct evidence of the model′s functional significance. Through these targeted studies, we can gradually translate current computational hypotheses into verifiable biological mechanisms.

## 5. Conclusion

In a nutshell, this computational study identified four genes—*BTBD6*, *C2CD4B*, *CCL24*, and *S100A4*—tied to macrophage/monocyte traits and UVM progression. The risk model based on these genes predicts survival and correlates with immune infiltration and drug sensitivity. But we lack direct evidence that these genes actively drive immune activity or tumor evasion. We point to a possible link between the four‐gene signature and immune modulation in UVM, yet functional work—especially on macrophage biology and immune pathways—is needed to back any mechanistic claims.

**Nomenclature**
UVM: uveal melanoma
WGCNA: weighted gene coexpression network analysis
TCGA: The Cancer Genome Atlas
GEO: Gene Expression Omnibus
scRNA‐Seq: single‐cell RNA sequencing
PCA: principal component analysis
DEGs: differentially expressed genes
GO: Gene Ontology
KEGG: Kyoto Encyclopedia of Genes and Genomes
ssGSEA: single‐sample gene set enrichment analysis
ROC: receiver operating characteristic curve
AUC: area under the curve
IC_50_
: inhibitory concentration in half

## Author Contributions

**Yuhui Yu:** experimental implementation, original manuscript drafting. **Mengya Wang:** supplementary molecular experiments, manuscript revision. **Bin Zhao:** research funding, overall project supervision, data integrity, conclusion accuracy. **Yuhui Yu** and **Mengya Wang** contributed equally to this work.

## Funding

No funding was received for this manuscript.

## Ethics Statement

Ethical approval was not required for this study because it did not involve any human experiments.

## Consent

The authors have nothing to report.

## Conflicts of Interest

The authors declare no conflicts of interest.

## Supporting Information

Additional supporting information can be found online in the Supporting Information section.

## Supporting information


**Supporting Information 1** Table S1: Sequences for liposome transfection.

**Supporting Information 2** Table S2: Primers for quantitative reverse transcription PCR.

## Data Availability

The datasets generated and/or analyzed during the current study are available in the GSE44295 repository (https://www.ncbi.nlm.nih.gov/geo/query/acc.cgi?acc=GSE44295) and the GSE139829 repository (https://www.ncbi.nlm.nih.gov/geo/query/acc.cgi?acc=GSE139829).
