# Supplementary material for: A Macrophage/Monocyte‐Related Four‐Gene Signature for Prognostic Assessment of Uveal Melanoma: BTBD6, C2CD4B, CCL24, and S100A4
Source: Hum Mutat. 2026 Jun 15;2026:4978880. doi: 10.1155/humu/4978880 (PMC13269842; doi:10.1155/humu/4978880)
Supplement: Supplementary file 1 — Supporting Information 1 Table S1: Sequences for liposome transfection. [file HUMU-2026-4978880-s001.docx]

Table S1. Sequences for liposome transfection

| Gene | Sequence (5’-3’) |
| --- | --- |
| si-C2CD4B#1 | CGTCTATTGGTAAAGAAGAAA |
| si-C2CD4B#2 | GATACTGCTTTATTGACGTTT |
| si-NC | GAGACAGAAATCTATTGGTAA |
