# Supplementary material for: A Macrophage/Monocyte‐Related Four‐Gene Signature for Prognostic Assessment of Uveal Melanoma: BTBD6, C2CD4B, CCL24, and S100A4
Source: Hum Mutat. 2026 Jun 15;2026:4978880. doi: 10.1155/humu/4978880 (PMC13269842; doi:10.1155/humu/4978880)
Supplement: Supplementary file 2 — Supporting Information 2 Table S2: Primers for quantitative reverse transcription PCR. [file HUMU-2026-4978880-s002.docx]

Table S2. Primers for quantitative reverse-transcription PCR

| Gene (Accession No) | Forward Primer (5’-3’) | Reverse Primer (5’-3’) |
| --- | --- | --- |
| BTBD6 (NM_033271) | GGAGACCTGGCGGAAGTCAAAT | AGCACCGTGTCGGCTTCCAGA |
| C2CD4B (NM_001007595) | AAGTGCTCACGCCGAATCGCAT | GCCACAGGTCGCTTTCAGCGG |
| CCL24 (NM_002991) | TGAGAACCGAGTGGTCAGCTAC | TTCTGCTTGGCGTCCAGGTTCT |
| S100A4 (NM_002961) | CAGAACTAAAGGAGCTGCTGACC | CTTGGAAGTCCACCTCGTTGTC |
| GAPDH (NM_002046) | GTCTCCTCTGACTTCAACAGCG | ACCACCCTGTTGCTGTAGCCAA |
